# Prostatic *Escherichia coli* infection drives CCR2-dependent recruitment of fibrocytes and collagen production

**DOI:** 10.1242/dmm.052012

**Published:** 2025-01-24

**Authors:** Brandon R. Scharpf, Hannah Ruetten, Jaskiran Sandhu, Kyle A. Wegner, Sneha Chandrashekar, Olivia Fox, Anne E. Turco, Clara Cole, Lisa M. Arendt, Douglas W. Strand, Chad M. Vezina

**Affiliations:** ^1^ Department of Comparative Biosciences, University of Wisconsin-Madison, Madison, WI 53706, USA; ^2^ George M. O'Brien Center for Benign Urologic Research, University of Wisconsin-Madison, Madison, WI 53706, USA; ^3^ Department of Urology, University of Texas Southwestern Medical Center, Dallas, TX 75390, USA

**Keywords:** Prostate, Fibrosis, Myofibroblast, Fibrocyte, LUTD, CCR2

## Abstract

Prostate fibrosis contributes to lower urinary tract dysfunction (LUTD). To develop targeted treatments for prostate fibrosis, it is necessary to identify the cell types and molecular pathways required for collagen production. We used a genetic approach to label and track potential collagen-producing cell lineages in mouse prostate through a round of *Escherichia coli* UTI89-mediated prostate inflammation. *E. coli* increased collagen density and production in *Gli1*^+^, *S100a4*^+^, *Lyz2*^+^ and *Cd2*^+^ cell lineages, but not in *Myh11*^+^ or *Srd5a2*^+^ cell lineages, in the mouse prostate. Molecular phenotyping revealed *GLI1*^+^*LYZ*^+^*S100A4^+^* cells (fibrocytes) in histologically inflamed human prostate. These fibrocytes colocalized with regions of increased collagen in men with LUTD. Fibrocyte recruitment and collagen synthesis was impaired in *Ccr2* null mice but restored by allotransplantation of Rosa-GFP donor bone marrow-derived cells. These results suggest that bone marrow-derived fibrocytes are a mediator of prostatic collagen accumulation.

## Teaser

**Summary:** This study identified CCR2-recruited fibrocytes that are necessary for collagen production in *E. coli*-mediated inflamed mouse prostates and a similar cell type in histologically inflamed human prostates.

## INTRODUCTION

Numerous studies have proposed a link between prostatic collagen accumulation and lower urinary tract dysfunction (LUTD) in men (Cantiello et al., 2013, 2014; Bauman et al., 2014; Zaitsev et al., 2016; Macoska et al., 2019). Rigidity of the prostate is associated with collagen content (Ma et al., 2012), which increases with age (Cantiello et al., 2013, 2014; Ruetten et al., 2018). Men with rigid prostate tissue are more likely to suffer from lower urinary tract symptoms, which can include frequent urination, nocturia, urgency, straining, intermittency, incomplete bladder emptying and/or a weak urinary stream (Rodriguez-Nieves and Macoska, 2013; Ma et al., 2012). The anatomical location of collagen accumulation in the aging male prostate has not been fully determined; however, it has been noted that collagen fibers do not accumulate in epithelial benign prostatic hyperplasia (BPH) nodules compared to histologically normal human prostate (Bauman et al., 2014). One goal of this study was to identify the anatomical location of collagen accumulation in the prostate of older men. Using a biobank of whole human prostate tissue specimens obtained by simple prostatectomy from men with LUTD and from young organ donors, we demonstrate that older men with LUTD (aged 65-82 years) have a thicker band of periurethral collagen than young men (aged 18-47 years).

The development of new therapies for prostate fibrosis necessitates an understanding of mechanisms and cell types mediating the disease. Prostate inflammation is a plausible driver of prostatic fibrosis in men (Cantiello et al., 2013), as it increases density of prostatic collagen fibers and collagen-positive cells, leading to urinary dysfunction (Wong et al., 2014; Ruetten et al., 2021; Bell-Cohn et al., 2019). Prostatic inflammation can be caused by many factors, including bacterial infection, age and genetics – all of which increase prostatic collagen deposition (Ruetten et al., 2021; Sciarra et al., 2007; Mizoguchi et al., 2017; Liu et al., 2019). Collagen-synthesizing cells associated with inflammation-mediated fibrosis across organs and tissues are diverse and include bone marrow-derived lymphoid and myeloid cells, perivascular fibroblasts (pericytes), fibroblasts and myofibroblasts (Zhang et al., 2019), but the precursor cell type(s) that contribute to inflammation-mediated prostate fibrosis are not fully known. A challenge in pinpointing inflammation-driven collagen-producing cell types is that inflammation can activate a cell-state change, altering the cellular phenotype in a way that makes the activated cell unrecognizable from its inactive precursor (Fang et al., 2018). Finding the precursor cell is important as it opens the possibility of targeting cell activation pathways for therapy, but is challenged by the heterogeneous and slow progression of benign prostatic disease in men.

The primary goal of this study was to identify precursor cell lineages that synthesize collagen in response to *Escherichia coli*-mediated prostate inflammation, a process that increases prostatic collagen content in mice (Ruetten et al., 2021). We deployed a genetic approach in mice to label and fate map cell types linked to fibrosis (myocytes, fibroblasts and circulating bone marrow-derived cells) to determine which cells expand and synthesize collagen in response to prostatic *E. coli* infection. We show that cells marked by alpha-smooth muscle actin (ACTA2) protein, a common identifier of myofibroblasts, are not collagen-producing cells in the *E. coli*-infected mouse prostate or in human BPH specimens. Cell lineage tracing supports the hypothesis that these cells are circulating myeloid-derived cells (*Lyz2*^+^*S100a4*^+^*Gli1*^+^) that home to *E. coli*-infected prostate by a (C-C motif) receptor 2 (CCR2)-dependent mechanism. We identified cells in regions of the inflamed human prostate that bear similar markers (*LYZ*^+^*GLI1*^+^*COL1A1*^+^) to fibrocytes in the *E. coli*-infected mouse prostate. Taken together, our results support a role for myeloid-derived fibrocytes in inflammation-mediated collagen production in the mouse prostate and support the identification of a similar population of cells in the human prostate.

## RESULTS

### The periurethral collagen band is thicker in men who had simple prostatectomy to treat LUTD than in young male organ donors

**Fig. 1.**
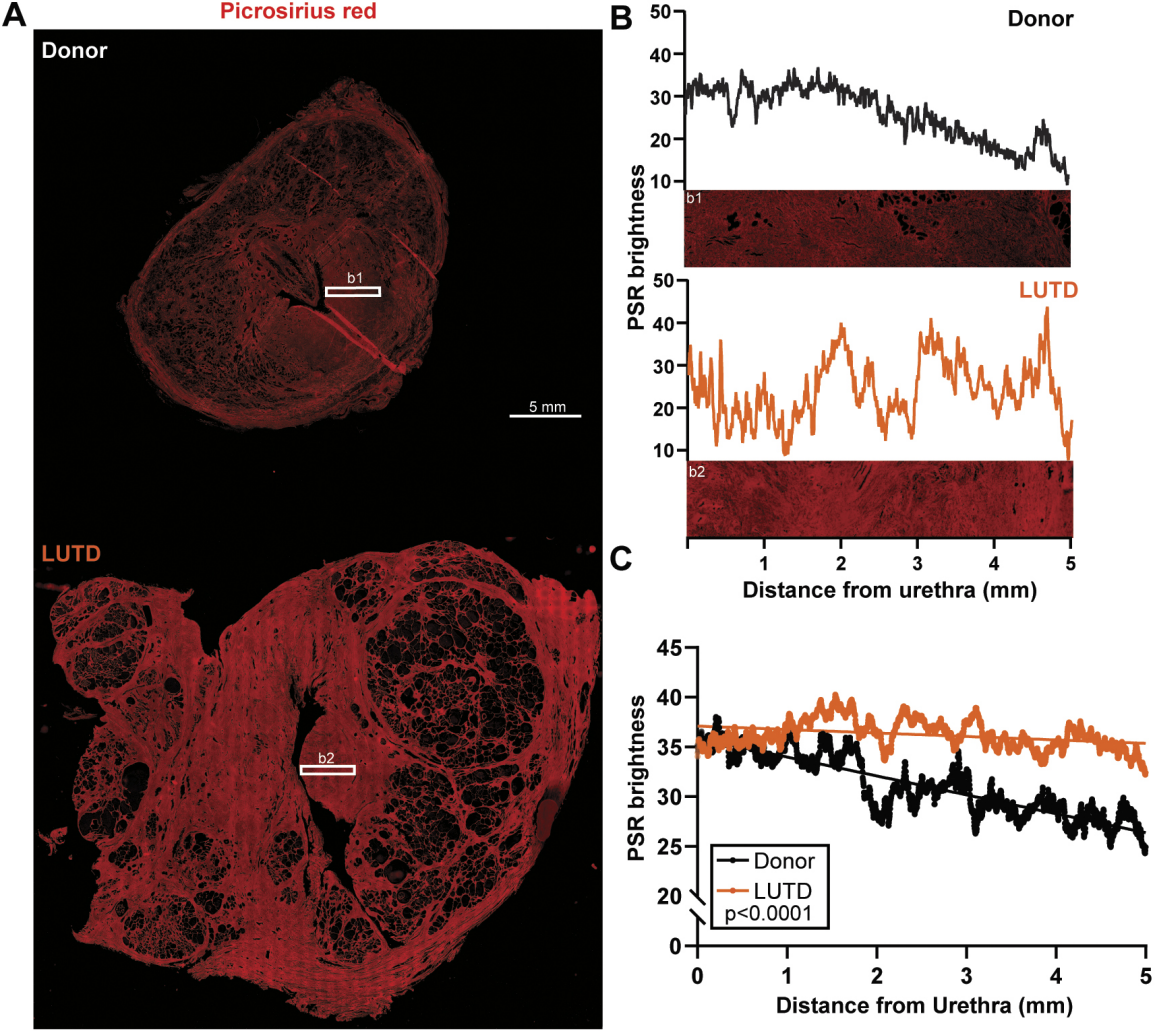
**The periurethral collagen band is thicker in men who had simple prostatectomy to treat lower urinary tract dysfunction (LUTD) than in young male organ donors.** Whole prostate sections were obtained from men aged 66-82 years who had their prostates removed by simple prostatectomy to treat LUTD (‘LUTD’) and from (young) male organ donors aged 18-47 years (‘Donor’)*.* (A) Prostate sections were stained with Picrosirius Red (PSR) and imaged using fluorescent microscopy. A 5.0 mm×1.0 mm region of interest (ROI) was selected adjacent to the center of the urethra, as indicated by a rectangle labeled 'b1' in the micrograph for the representative donor prostate and 'b2' in the micrograph for the LUTD prostate. Scale bar: 5 mm. (B) Representative plots showing brightness of PSR stain versus distance from the urethra in an ROI from one donor and one LUTD sample. (C) Average PSR brightness across all prostate samples. Donor samples have abundant PSR stain immediately adjacent to the urethra that decreases with distance from the urethral edge. LUTD patient prostates maintain significantly greater PSR stain abundance with distance from urethra. Results are shown from ten donor and 17 LUTD prostates. Linear regression analysis was performed to determine the coefficient of determination (*r*^2^) and whether slopes of linear regression lines significantly differed (*P*<0.05).

We previously reported the presence of a dense collagen band in the prostate periurethral region of rodents, canines and young healthy men (Ruetten et al., 2018). Here, we tested whether periurethral collagen is thicker in older men with LUTD than in young men. Transverse prostate sections were prepared from 17 male patients (Table S1) who underwent simple prostatectomy to treat LUTD (hereafter referred to as LUTD prostates) and from ten organ donors across a wide age range who did not have their prostates removed owing to LUTD (hereafter referred to as donor prostates). We used Picrosirius Red (PSR) to stain collagen and visualized collagen fibers under fluorescent light (Fig. 1A). PSR collagen staining was evident throughout the prostate gland and was noticeably dense in the periurethral region and surrounding proximal prostate ducts. We quantified PSR pixel density as a function of distance from the urethra using the method outlined in Fig. S1 (Fig. 1B). The PSR staining in LUTD prostates was denser, and pixels were extended further away from the urethra, than in donor prostates (Fig. 1C).

### Collagen-producing (proCOL1^+^) cells in the inflamed mouse and human prostate are ACTA2^−^ (non-myofibroblasts)

**Fig. 2.**
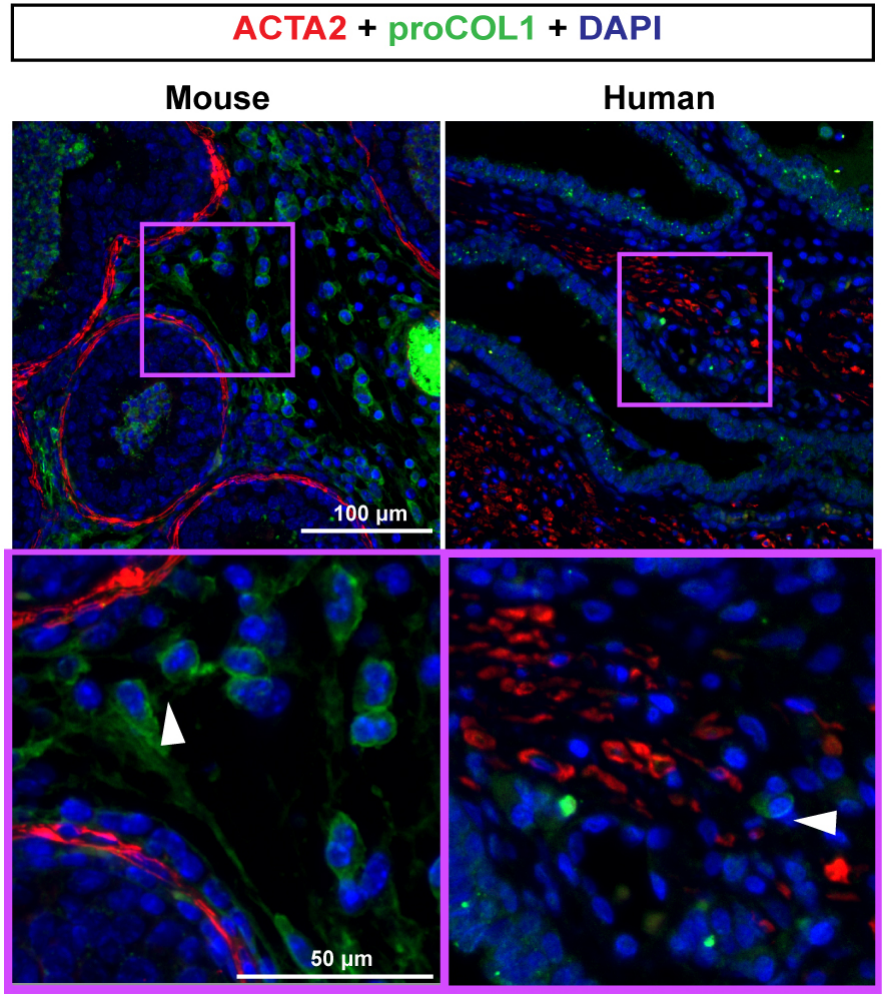
**Collagen-producing (proCOL1^+^) cells in the inflamed mouse and human prostate are ACTA2^−^ (non-myofibroblasts).** Human prostate sections were obtained as described in the Materials and Methods. Eight-week-old WT mice were transurethrally catheterized and administered two bolus doses, spaced 24 h apart, of *E. coli* UTI89 [optical density (OD) 0.80 in 100 µl PBS]. Mice were euthanized 7 days after the first bolus dose of *E. coli*. Lower urinary tracts were collected, fixed, embedded in paraffin and sectioned. Inflamed mouse and human prostate samples were immunostained with antibodies against alpha-smooth muscle actin (ACTA2) and procollagen 1 (proCOL1). Nuclei were stained with DAPI. Arrowheads indicate ACTA2^−^, proCOL1^+^ cells. Images were taken using an Eclipse E600 compound microscope at 20× magnification. Results are representative of three individuals per group. Scale bars: 50 µm (bottom) and 100 µm (top).

Myofibroblasts, characterized by ACTA2 and collagen co-expression, contribute to fibrosis in lung, heart, kidney, liver and skin (Phan, 2012; Wynn, 2008; Peyser et al., 2019; Hinz et al., 2012). TGFB1 or CXCL12 drive isolated primary prostate fibroblasts to co-express ACTA2 and COL1A1 in culture, suggesting the potential for prostate fibroblasts to transition to myofibroblasts under some conditions (Gharaee-Kermani et al., 2012). However, under the condition of *E. coli* infection, ACTA2 and procollagen 1 (proCOL1) co-expressing cells are rare in the mouse and canine prostate (Ruetten et al., 2021), prompting us to test whether proCOL1 and ACTA2 co-expressing cells are also rare in histologically inflamed human prostate. Immunofluorescence (IF) revealed that most proCOL1^+^ cells were ACTA2^−^ in inflamed human and mouse prostate (Fig. 2). We used CD45^+^ (also known as PTPRC^+^) leukocyte density as an index of inflammation for human prostate tissue. Inflamed human samples are further shown in Fig. S2.

### Prostate inflammation expands *Gli1*^+^, *Lyz2*^+^, *Cd2*^+^ and *S100a4*^+^ cell lineages, but not *Srd5a2*^+^ or *Myh11*^+^ cell lineages

**Fig. 3.**
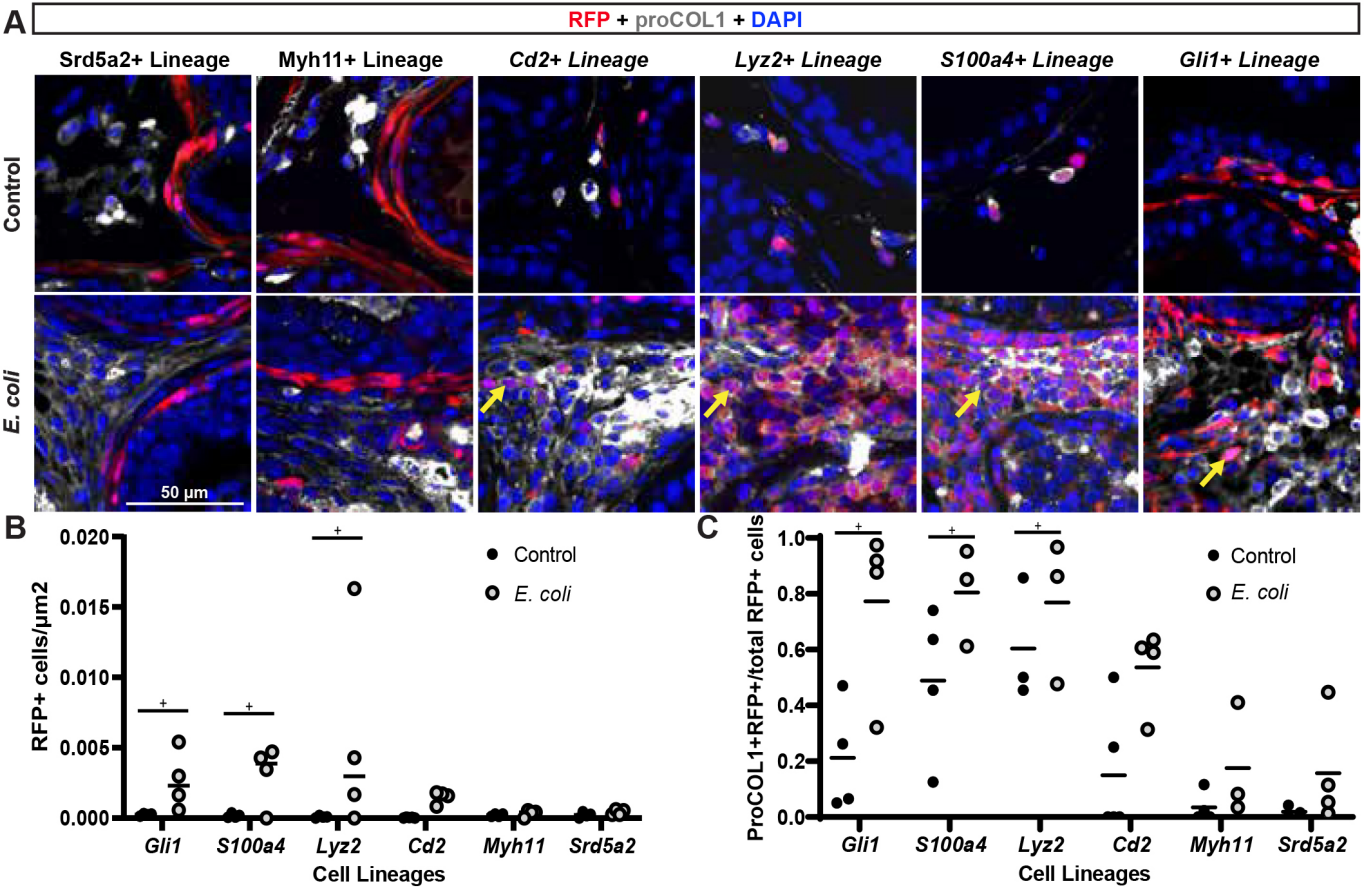
**Prostate inflammation expands *Gli1*^+^, *Lyz2*^+^, *Cd2*^+^ and *S100a4*^+^ cell lineages but not *Srd5a2*^+^ or *Myh11*^+^ cell lineages.** The Ai14 reporter allele was bred to each of the following strains to create reporter mice: *Cd2-icre*, *Gli1tm3(cre/ERT2*), *Lyz2tm1(cre)*, *Myh11-cre*, *S100a4-cre* and *Srd5a2-G2aCE.* Seven-week-old *Gli1* and *Srd5a2* reporter mice were administered 200 mg/kg/day of tamoxifen for 4 days to activate *cre*; the other *cre* alleles are constitutively active. At 8 weeks of age, reporter mice were transurethrally catheterized and administered two bolus doses, spaced 24 h apart, of *E. coli* UTI89 (OD 0.80 in 100 µl PBS) or sterile PBS. Mice were euthanized 7 days after the first bolus dose of *E. coli*. (A) Lower urinary tracts were collected, fixed, embedded in paraffin, sectioned and immunostained with antibodies against proCOL1 and RFP to visualize the Ai14 reporter, and with DAPI to visualize nuclei. Yellow arrows indicate the locations of RFP^+^ and proCOL1^+^ co-expressing cells. Images were taken using an Eclipse E600 compound microscope at 20× magnification. Scale bar: 50 µm. (B,C) Densities (B) and proportions (C) of RFP^+^ cells that produce collagen were determined. Results show three to five mice per group. Individual lineages were compared to their own respective control using a paired two-tailed Student's *t*-test. ‘+’ indicates a significant difference (*P*<0.05) from the control group.

We utilized a genetic strategy to label and fate map multiple cell types through a round of *E. coli-*induced prostate infection/inflammation in mice. The Ai14 reporter allele, encoding a modified cre-inducible RFP, was bred to each of the following to create reporter mice carrying one copy of each allele: *Cd2-cre*, *Gli1-creER^T2^*, *Lyz2-creER^T2^*, *Myh11-cre*, *S100a4-cre* and *Srd5a2-creER^T2^. Cd2* marks lymphoid cells (de Boer et al., 2003); *Gli1* marks pericytes and prostate periductal stromal cells with progenitor characteristics (Peng et al., 2013; Kramann et al., 2015); *Lyz2* was described previously as a marker of myeloid cells (Canli et al., 2017); *Myh11* was described previously as a marker of prostatic smooth muscle myocytes (Welsh et al., 2011) and myofibroblasts (Karthaus et al., 2020); and *S100a4* and *Srd5a2* were shown to mark prostate ductal fibroblasts (Bhowmick et al., 2004; Wegner et al., 2017a; Joseph et al., 2021). To induce prostatic inflammation, 8-week-old (adult) male reporter mice were anesthetized, transurethrally catheterized, and instilled with either sterile PBS or *E. coli* UTI89, a uropathogen known to drive prostatic inflammation and collagen synthesis, and increase collagen-producing cell density (Ruetten et al., 2021). Prostate tissue was collected 1 week after catheterization. *E. coli* significantly increased the prostatic RFP^+^ cell density and the proportion of RFP^+^ cells co-expressing proCOL1 in mice of *Cd2*^+^, *Gli1*^+^, *Lyz2*^+^ and *S100a4*^+^ cell lineages without significantly changing these cell densities in mice of *Myh11*^+^ and *Srd5a2*^+^ cell lineages (Fig. 3A-C). We used Ki67 (also known as MKI67) to identify cells in the proliferative phase of the cell cycle and determine whether cell lineages are expanding because they are recruited *de novo* (Ki67^−^) or are proliferating (Ki67^+^) in the *E. coli*-infected prostate. *E. coli* infection increased the proportion of RFP^+^ cells that co-express Ki67 in mice of *Lyz2*^+^ and *Gli1*^+^ cell lineages, but not in mice of *Cd2*^+^ and *S100a4*^+^ cell lineages (Fig. S3).

### Molecular phenotyping of two distinct mouse strains demonstrates the presence of *Gli1*, *Lyz2* and *S100a4* triple-positive cells in *E. coli*-infected mouse dorsal prostate

**Fig. 4.**
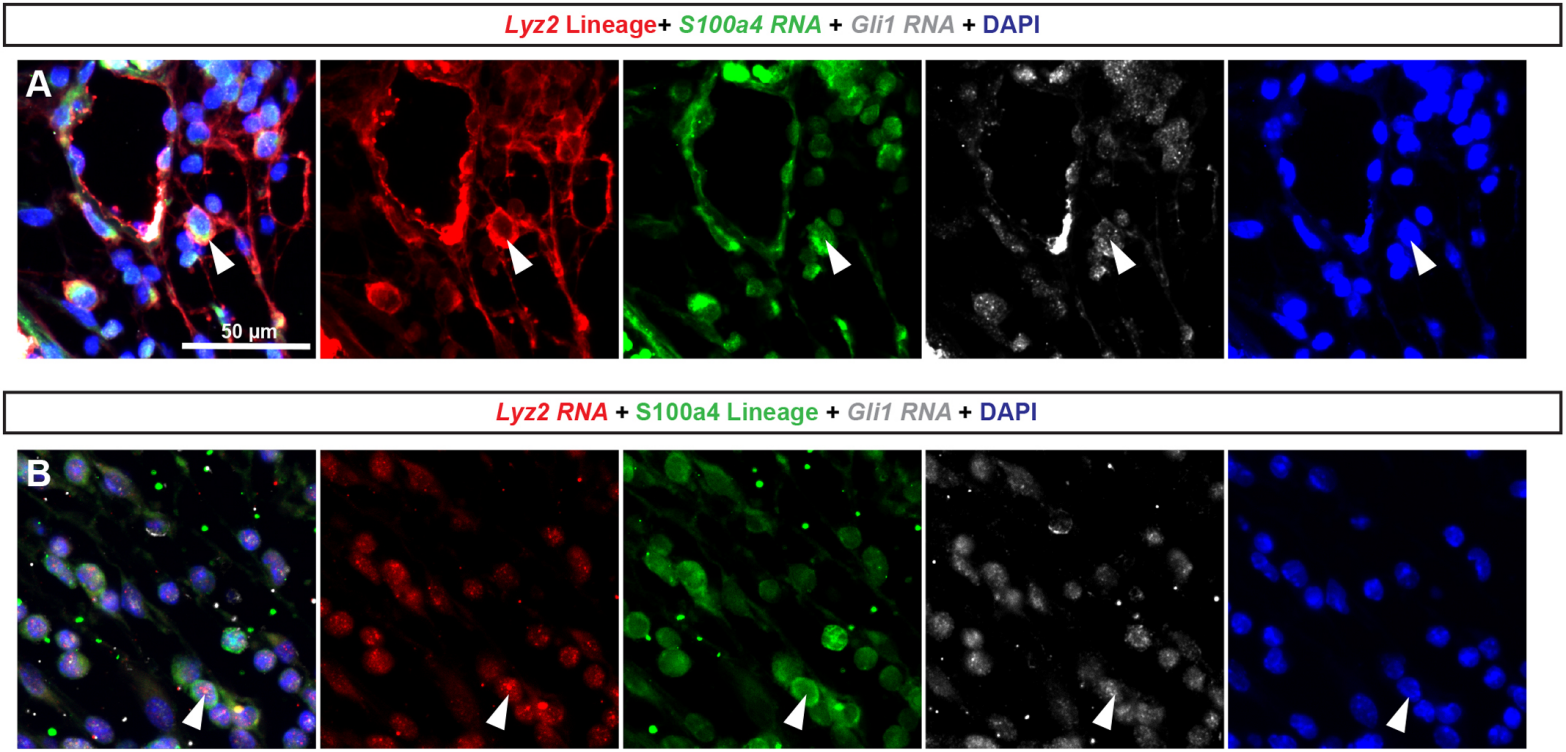
**Molecular phenotyping two distinct mouse strains demonstrates the presence of *Gli1*, *Lyz2* and *S100a4* triple-positive cells in *E. coli*-infected mouse dorsal prostate.** Eight-week-old male mice were anesthetized and *E. coli* (100 µl; OD 0.8) was introduced via a transurethral catheter. Prostate tissue was collected 1 week later, fixed and paraffin embedded. (A*) Lyz2tm1*(*cre*); R26R-tdtomato mouse dorsal prostate sections were labeled with hybridization chain reaction (HCR) probes against *Gli1* and *S100a4*, and immunolabeled with an antibody against RFP. (B) *S100a4-cre*; R26R-tdtomato mouse dorsal prostate sections were labeled with HCR probes against *Lyz2* and *Gli1* mRNA, and immunolabeled with an antibody against RFP. Arrowheads indicate triple-positive cells. Images were taken using an Eclipse E600 compound microscope at 20× magnification. Results are representative of three mice per group. Scale bar: 50 µm.

We calculated the percentage contribution of each fate-mapped cell population to the total population of collagen-producing cells in the inflamed dorsal prostate. *S100a4^+^* fate-mapped cells represented ∼86% of collagen-producing cells, while *Lyz2+* and *Gli1+* fate-mapped cells each represented 70% of collagen-producing cells, in the dorsal prostate (Table S2). The individual contributions of *Gli1^+^*, *Lyz2^+^* and *S100a4^+^* lineages to prostatic collagen-producing cells exceeded 100%, suggesting intersection among the cell lineages. A closer examination using hybridization chain reaction, a multiplex RNA detection method, revealed the co-expression of *Gli1*, *Lyz2* and *S100a4* mRNA in some cells of the *E. coli*-infected mouse prostate (Fig. 4). As further evidence of overlap among *Gli1*^+^, *Lyz2*^+^ and *S100a4*^+^ cell lineages, we showed that RFP^+^ fate-mapped cells from the *Lyz2^+^* lineage expressed *Gli1* and *S100a4* mRNA, and fate-mapped RFP^+^ cells from the *S100a4*^+^ lineage expressed *Lyz2* and *Gli1* mRNA (Fig. 4A,B).

### *LYZ*^+^/*GLI1*^+^/S100A4^+^ triple-positive cells are present in the transition zone of the histologically inflamed human prostate and produce collagen

**Fig. 5.**
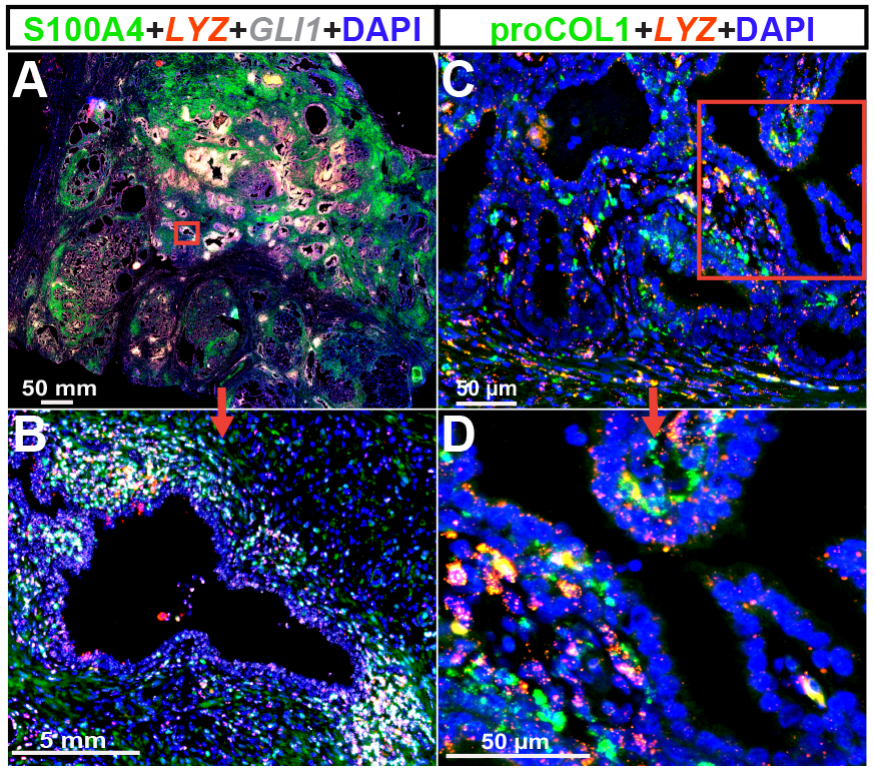
***LYZ*^+^/*GLI1*^+^/S100A4^+^ triple-positive cells are present in the transition zone of the inflamed human prostate and produce collagen.** Human prostate samples, obtained by simple prostatectomy from men with LUTD (Table S1), were cut into sections and categorized as having high-grade inflammation based on the total number of CD45^+^ immune cells (Fig. S2). RNAscope™ assay was used to visualize *LYZ* and *GLI1* mRNA, and immunofluorescence (IF) was used to visualize S100A4 and proCOL1 protein. Tissues were imaged using a Keyence BZ-X700 microscope, with images stitched and compressed into whole images [magnification at 4× (A), 10× (B) or 20× (C)], or an Eclipse E600 compound microscope at 20× magnification (D; Fig. S4). Scale bars: 50 mm (A,C,D) and 5 mm (B). Results are representative of five individuals per group.

Resident and non-resident cells have been implicated in organ fibrosis, and, in some cases, multiple cell types contribute to collagen synthesis in fibrotic tissues (Tsukui et al., 2020; Forbes et al., 2004). To determine whether the histologically inflamed human prostate harbors cells that are phenotypically similar to the collagen-producing cells that accumulate in the *E. coli*-infected mouse prostate, we utilized fluorescent RNAscope^®^ with probes against *GLI1* and *LYZ*, and IF with antibodies against S100A4. In histologically inflamed regions, we identified a population of cells that co-express *GLI1*, *LYZ* and S100A4 within the human prostate transition zone (Fig. 5A,B; Fig. S4). Triple-positive cells identified in human prostate support our results in mouse prostate. Additionally, *LYZ*^+^ cells were shown to produce collagen in pockets of high inflammation (Fig. 5C,D).

### *Ccr2* is required for the recruitment and infiltration of collagen-producing cells to *E. coli*-infected mouse prostate and the fibrotic response to prostate inflammation

**Fig. 6.**
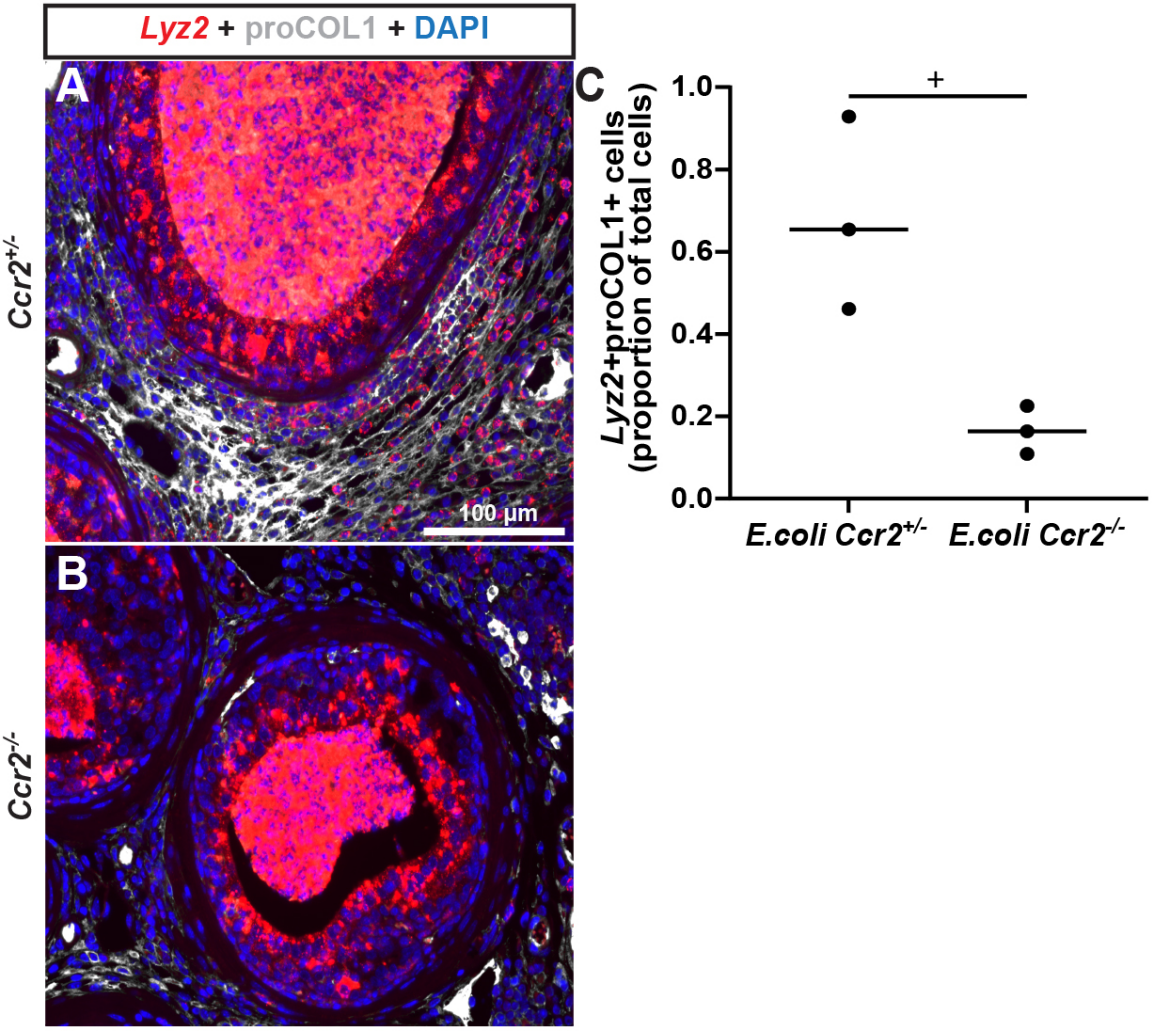
***Ccr2* is required for recruitment and infiltration of collagen-producing cells to *E. coli*-infected mouse prostate.** Eight-week-old control (*Ccr2^+/−^*) and *Ccr2* null (*Ccr2^−/−^*) male mice were transurethrally catheterized and administered two bolus doses, spaced 24 h apart, of *E. coli* UTI89 (OD 0.80 in 100 µl PBS) or sterile PBS. Mice were euthanized 7 days after the first bolus dose of *E. coli*. (A,B) *Ccr2*^+/−^ (A) and *Ccr2*^−/−^ (B) mouse dorsal prostate sections were stained with an antibody against the intracellular collagen precursor, proCOL1. Images were taken in the dorsal prostate lobe using an Eclipse E600 compound microscope at 20× magnification. Scale bar: 100 µm. Individual channels are provided in Fig. S5 for further emphasis of co-expression. (C) RNAscope™ assay was used to visualize *Lyz2* mRNA. *Lyz2*^+^proCOL1^+^ double-positive cells were significantly less frequent in *Ccr2*^−/−^ than in genetic control *Ccr2*^+/−^mouse prostates (three mice per experimental group). Groups were compared using an unpaired two-tailed Student's *t*-test. ‘+’ indicates a significant difference (*P*<0.05) between groups.

**Fig. 7.**
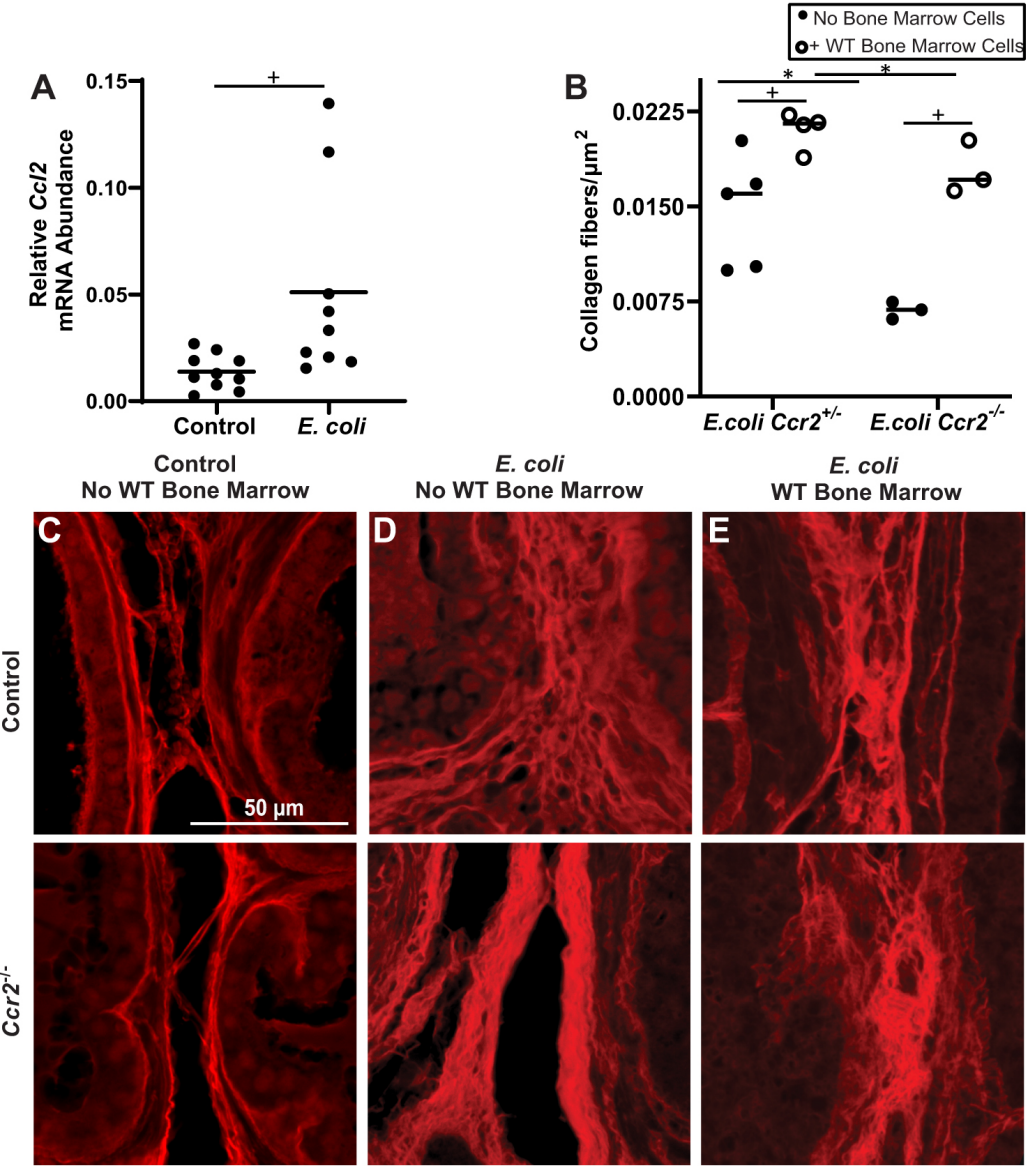
***Ccr2*+ circulating bone marrow-derived cells drive the fibrotic response to prostate inflammation.** Eight-week-old control (*Ccr2^+/−^*) and *Ccr2* null (*Ccr2^−/−^*) male mice were anesthetized with isoflurane and retro-orbitally injected with bone marrow cells from Rosa-GFP donor mice (27G needle, 5 million cells in 100 µl PBS) or PBS alone. Mice were then immediately transurethrally, catheterized and administered the first of two bolus doses, spaced 24 h apart, of *E. coli* UTI89 (OD 0.80 in 100 µl PBS) or sterile PBS. Eight-week-old wild-type mice were similarly infected with *E. coli* UTI89. Mice were euthanized 7 days after the first bolus dose of *E. coli*. (A) The expression of C-C motif chemokine ligand 2 (*Ccl2*) mRNA, which encodes the protein ligand of CCR2, was measured in dorsal prostate and normalized to that of *Ppia*. (C-E) Lower urinary tracts of *Ccr2^−/+^* and *Ccr2^−/−^* male mice were collected, fixed, embedded in paraffin, sectioned, stained with PSR, illuminated, and imaged using a Texas Red filter to reveal collagen fibers on an Eclipse E600 compound microscope at 20× magnification. Scale bar: 50 µm. (B) Collagen fibers per image were quantified with CT-FIRE software. Results are from nine to ten (A) or three to five (B-E) mice per group. Groups were compared using an unpaired two-tailed Student's *t*-test (A) or a two-way ANOVA that passed (*P*>0.05) Spearman's test for heteroscedasticity and the Shapiro–Wilk test for normality of residuals followed by Sidak's multiple comparisons test (B). Treatment (*P=0.0002) and genotype (^+^*P*=0.004) had significant effects; interaction was not significant. *P*<0.05 was considered statistically significant.

Molecular phenotyping of collagen-producing cells in the *E. coli*-infected mouse prostate, most notably the expression of *Lyz2* and CD45, suggests a myeloid lineage, raising the possibility that these cells initiate as circulating fibrocytes and home to inflamed prostate tissue to initiate collagen synthesis. Myeloid-derived fibrocytes are implicated in fibrosis of lung and colon (which share some structural and physiological characteristics with prostate, as all three tissues derive from endoderm) (Andersson-Sjöland et al., 2008; Kuroda et al., 2019). Circulating fibrocytes express the chemokine CCR2, which is activated by C-C motif chemokine ligand 2 (CCL2) secreted from damaged tissue to drive fibrocyte recruitment (Moore et al., 2001, 2005; Kuroda et al., 2019). We found that *E. coli* infection of the mouse prostate increased *Lyz2*^+^ cell abundance (Fig. 6; Fig. S5) and expression of *Ccl2* mRNA (Fig. 7A). Using *Ccr2^+/−^* mice, which carry one functional *Ccr2* allele and one RFP knock-in allele, which replaces the *Ccr2* coding region (Saederup et al., 2010), we also found that *E. coli* infection increased prostatic *Ccr2^+^* cell abundance (Fig. S6). To test whether *Ccr2* is required for the fibrotic response to prostatic *E. coli* infection, *Ccr2^−/−^* (*Ccr2* null) and *Ccr2^+/−^* (control) mice were transurethrally instilled with sterile PBS or *E. coli*. We used CD45^+^ leukocyte density as an index of inflammation and found that *E. coli* drives a comparable inflammatory response in the prostates of control and *Ccr2* null mice (Fig. S7). PSR staining of the resulting prostate tissue sections was used to visualize collagen, revealing that *E. coli* increased prostatic collagen abundance in *Ccr2*^+/−^ genetic control, but not *Ccr2* null, mice (Fig. 7C,D). We conducted an allograft experiment to test, specifically, whether circulating CCR2^+^ cells mediate the fibrotic response to prostate inflammation. We collected femur bone marrow cells from Rosa-GFP mice, injected them into the retro-orbital sinus of control and *Ccr2* null mice, and delivered transurethral *E. coli* to drive prostate inflammation (Fig. S8). Allotransplantation of Rosa-GFP marrow cells into *Ccr2* null mice restored the fibrotic response to inflammation (Fig. 7B-E).

## DISCUSSION

Elucidating the cell types responsible for prostate fibrosis is a step towards the development of therapies targeting the profibrotic pathways uniquely activated in those cells. Previous studies identified molecular pathways that drive the phenoconversion of prostate fibroblasts to collagen-producing myofibroblasts (Hata et al., 2020; Sheng et al., 2018), but ACTA2^+^ myofibroblasts are rare in mouse and human prostate. We used a cell lineage tracing approach to indelibly label a variety of cells and observe cell behaviors through a round of prostatic *E. coli* infection, which drives inflammation and collagen accumulation in the mouse prostate. *Gli1*^+^*S100a4*^+^*Lyz2*^+^ cells home to the inflamed prostate, expand and produce collagen (Figs 3 and 6), and we detected a phenotypically similar cell type in inflamed human prostate tissue sections (Fig. 5). Our results support the hypothesis that bone marrow-derived fibrocytes are responsible for inflammation-mediated collagen production in the mouse prostate.

CCR2 is required for the accumulation of collagen-producing cells in the *E. coli*-inflamed mouse prostate, and allotransportation of Rosa-GFP donor cells into *Ccr2* null mice is sufficient to restore *E. coli*-induced prostate collagen accumulation to the *E. coli*-infected mouse prostate. CCR2 antagonists demonstrate promise in preclinical models of metabolic dysfunction-associated steatohepatitis and renal fibrosis (Miao et al., 2016; Lefere et al., 2020; He et al., 2023). CCR2 antagonists can block monocyte infiltration, halt inflammatory cell activation and, consequently, reduce inflammation (Miao et al., 2016; Lefere et al., 2020; He et al., 2023). Additionally, CCR2 antagonists could prove to be even more potent when used in combination with other drugs, like an FGF21 analog, which further reduces fibrosis in rodents (Puengel et al., 2022). CCR2 agonists have multimodal mechanisms of action; in addition to blocking the migration of fibrocytes into inflamed tissues, CCR2 antagonists can inhibit the infiltration and activation of profibrotic macrophages (Guo et al., 2023; Peng et al., 2022). In the current study, *E. coli* infection of *Ccr2* null mouse prostate did not inhibit leukocyte migration into the prostate, as the CD45^+^ cell density did not differ between wild-type and *Ccr2* null mice (Fig. S7). Instead, genetic deletion of *Ccr2* significantly reduced the density of proCOL1-positive cells in the *E. coli*-infected prostate, suggesting that resistance of *Ccr2* null mice to *E. coli*-mediated prostate collagen accumulation is due to a defect in fibrocyte migration. Previous studies have shown that CCR2 is required for fibrocyte migration into the inflamed colon, lung and kidney (Moore et al., 2005; Kuroda et al., 2019; Reich et al., 2013). Although CCR2 has been targeted experimentally in models of prostate cancer (Loberg et al., 2007; Kirk et al., 2013), the impact of CCR2 blockade on human prostate fibrosis has not been examined.

We identified phenotypically similar cells in the *E. coli*-infected mouse prostate and the histologically inflamed human prostate, but whether fibrocyte recruitment to the prostate is a generalizable mechanism for all forms of human prostate fibrosis is not known. Fibroblasts are unique in that they phenotypically differ among organs and can perform multiple roles within single organs (Zeisberg and Kalluri, 2013). Interestingly, the collagen-producing cell type in some organs with a particular fibrosis-induced condition can be dependent on activated fibroblasts. For example, oxidative stress/TGFB1 activates intrapulmonary myofibroblasts in idiopathic pulmonary fibrosis, and bleomycin recruits bone marrow-derived fibrocytes in bleomycin-induced pulmonary fibrosis (Zeisberg and Kalluri, 2013; Hashimoto et al., 2004; Todd et al., 2012). Whether the molecular phenotype of prostate recruited myeloid cells (expression of *Gli1*, *Lyz2*, *S100a4*) and other markers changes over time, especially after resolution of *E. coli* infection, was not examined in this study. Owing to the limitations of our collagen quantification method, the percentage of PSR-stained collagen fibers derived from fibrocytes in the *E. coli*-inflamed mouse prostate was not examined.

A variety of mechanisms have been linked to prostate inflammation. We used a common gram-negative uropathogen to drive prostate inflammation in this study, but other pathogens are linked to prostate inflammation in men (Alluri et al., 2021; Karami et al., 2022). There is growing evidence that autoimmune inflammation of the prostate is a mechanism of urinary voiding dysfunction in humans (Vickman et al., 2022), and autoimmune inflammation of the mouse prostate increases prostatic collagen density (Roman et al., 2016). Parasites may also contribute to prostate inflammation (Colinot et al., 2017). Whether all prostate inflammation mechanisms cause fibrosis, and whether the fibrotic mechanism involves fibrocytes or another cell type, is not known. The persistence of collagen synthesized in response to *E. coli* infection has been shown (Ruetten et al., 2023), but the persistence of fibrocytes recruited to the *E. coli*-infected prostate was not examined in this study. We are planning to address these in our future studies by investigating how recruited fibrocytes, after a single round of *E. coli* infection, respond to subsequent infections.

In conclusion, we used a cell lineage tracing approach involving six different cre-expressing mouse strains and present evidence that a circulating myeloid-derived fibrocyte is recruited to the *E. coli*-infected mouse prostate by a CCR2-dependent mechanism to synthesize collagen to increase prostatic collagen density. We identified a phenotypically similar cell in the human prostate and suggest that fibrocytes are a mediator of prostatic collagen accumulation in response to bacterial infection and possibly other fibrotic mechanisms.

## MATERIALS AND METHODS

### Human tissues

Prostate specimens from organ donors whose families consented at the Southwest Transplant Alliance under Institutional Review Board STU 112014-033 were collected by cystoprostatectomy after collection of transplantable organs and are referred to as ‘donor’ samples. Prostate specimens from patients seeking treatment for LUTD were collected by simple prostatectomy at the University of Texas Southwestern and are referred to as ‘LUTD’ or ‘BPH’ samples. Tissue samples (>24 h post-mortem) were transported in ice-cold saline and dissected into portions for fixation in 10% formalin followed by paraffin embedding. Information on human specimens is provided in Table S1. All clinical investigation was conducted according to the principles expressed in the Declaration of Helsinki.

### Mice

All experiments were conducted under an approved protocol from the University of Wisconsin Animal Care and Use Committee and in accordance with the National Institutes of Health Guide for the Care and Use of Laboratory Animals. Mice were housed in Udel^®^ Polysulfone microisolator cages on racks or in Innocage^®^ disposable mouse cages on an Innorack^®^; room lighting was maintained on 12 h light and dark cycles; room temperature was typically 20.5±5°C; humidity was 30-70%. Mice were fed 8604 Teklad Rodent Diet (Harlan Laboratories, Madison, WI, USA), and feed and water were available *ad libitum*. Cages contained corn cob bedding.

Mice were purchased from The Jackson Laboratory (Bar Harbor, ME, USA) and backcrossed onto C57BL/6J background for four to six generations. Male mice used in studies were aged 8-10 weeks. Stock numbers included the following: *B6.129(C*g*)*-*Ccr2tm2.1Ifc/J* (Stock #017586) (Saederup et al., 2010), *Gli1tm3(cre/ERT2)*Alj/J (Stock #007913) (Ahn and Joyner, 2004), *BALB/c-*Tg(*S100a4-cre*)1Egn/YunkJ (Stock #012641) (Tsutsumi et al., 2009), *Srd5a2-G2aCE* (Stock #028117) (Wegner et al., 2017a), *B6.Cg-Tg(Myh11-cre,-EGFP)2Mik/J* (Stock #007742) (Xin et al., 2002), *B6.Cg-Tg(Cd2-icre)4KioJ* (Stock #008520) (de Boer et al., 2003), B6.129P2-Lyz2tm1(cre)IfoJ (Stock #004781) (Clausen et al., 1999), Rosa *R26R-tdTomato* (Stock #007914) (Madisen et al., 2010) and B6.Cg-Tg(Gt(ROSA)26Sor-EGFP)I1Able/J (Stock Number #007897) (Giel-Moloney et al., 2007).

### Mouse prostate inflammation

Uropathogenic *E. coli* (UPEC) was used to drive prostate inflammation in mice. *E. coli* UTI89 (Mysorekar and Hultgren, 2006) is a strain of uropathogenic *E. coli* recovered from a human patient with cystitis (Langermann et al., 2000) and was previously transformed with a pCOMGFP plasmid (Valdivia et al., 1996), conferring green fluorescent protein (GFP) expression and kanamycin resistance. Prior to inoculation, *E. coli* UTI189 was grown as a static culture at 37°C for 18 h in antibiotic free Luria-Bertani broth. The optical density (OD) was determined prior to mouse inoculation. The culture was centrifuged for 15 min at 11.3 ***g*** and the resulting pellet was resuspended in sterile phosphate-buffered saline (PBS) for instillation. Mice were anesthetized with isoflurane and then transurethrally catheterized and administered two bolus doses, spaced 24 h apart, of 100 µl sterile PBS (uninfected control) or PBS containing *E. coli* UTI189 (OD 0.80, via transurethral catheter as previously described; Ruetten et al., 2021).

### Allotransplant of bone marrow cells into mice

Bone marrow was collected from Rosa-GFP donor mice. The mice were euthanized by CO_2_ asphyxiation and dipped in 70% ethanol. An incision was made in the skin overlying the tibia, femur and humerus, and muscles were trimmed away to expose the bones. The bones were disarticulated, cleaned of muscle and other exterior tissue, and transferred to ice-cold 1× PBS. The epiphyses were cut at each end, creating holes to insert a 27G needle. The needle was inserted into the end of the bone, and the bone marrow was flushed with Hank's balanced salt solution (HBSS) into a collection tube. The process was repeated from the other end of the bone until the bone appeared clear and free of bone marrow. The cell solution (bone marrow and HBSS) was gently pipetted to disperse cell clumps and centrifuged for 10 min at 300 ***g***. The supernatant was removed, and cells were resuspended in 1 ml red blood cell lysis buffer for 1-2 min. The solution was diluted in 15 ml HBSS and centrifuged for 10 min at 300 ***g***. The supernatant was removed, and the cells were suspended in 1 ml HBSS for cell counting. Live-cell counting was performed by adding 100 µl cells to a 1 ml tube with 400 µl Trypan Blue (0.4%) and applying 100 µl of the cell suspension to a hemocytometer. Cell suspension was centrifuged at 300 ***g*** for 10 min, and cells were resuspended in 1× PBS (5 million cells per 100 µl). Eight-week-old male mice carrying one (control) or two copies of the *Ccr2^tm2.1^* allele received a retroorbital injection of 100 µl PBS containing 5 million bone marrow cells from Rosa-GFP donor mice or equal parts saline 1-2 min before the intraurethral instillation of *E. coli* UTI89. IF was performed to confirm the migration of Rosa-GFP donor cells into the *E. coli*-inflamed mouse prostate (Fig. S8).

### Histology and immunostaining

Lower urinary tracts were collected for histology 7 days after transurethral instillation. Tissues were prepared, fixed and sectioned as described previously (Mehta et al., 2011; Wegner et al., 2019; Wegner and Vezina, 2019). To remove the lower urinary tract, ureters were cut at the entry to the bladder wall, the vas deferens was cut at the entry to the bladder neck, and the urethra was cut immediately dorsal to the pubic symphysis. Tissues were fixed in the sagittal plane, dehydrated in ethanol, cleared in xylene and infiltrated with paraffin. Dorsal prostate sections (5 μm) were cut and mounted on Superfrost™ Plus Gold Slides (Thermo Fisher Scientific, Waltham, MA, USA).

IF was conducted on dorsal prostate tissue sections. Tissue sections were deparaffinized with xylene and rehydrated through a graded ethanol series. The tissues were immersed in citrate buffer pH 6.0 heated in a microwave for epitope decloaking. Tris-buffered saline containing 0.1% Tween 20 and 5% donkey serum was used as a blocking reagent, and primary and secondary antibodies were diluted in blocking reagent. Antibodies and dilutions are shown in Table S3. Nuclei were stained with 2-(4-amidinophenyl)-1H-indole-6-carboxamidine (DAPI). Tissue sections (inflammation pockets of dorsal lobe for mice, periurethral region for human) were imaged using an Eclipse E600 compound microscope (Nikon Instruments, Melville, NY, USA) fitted with a 20× dry objective (Plan Fluor, 0.75 NA; Nikon Instruments) and equipped with NIS elements imaging software (Nikon Instruments) and FITC, Texas Red (Chroma Technology Corp, Bellows Fall, VT, USA), and CY5 filter cubes (Nikon Instruments). All nucleated cells outside of blood vessels and the prostate ductal lumen were manually counted using ImageJ cell counter (Schindelin et al., 2012). All partially or completely stained cells were considered positive.

### PSR staining and collagen quantification

Mouse and human tissue sections were stained with PSR as described in a method that we previously published, which enhances its sensitivity (Wegner et al., 2017b). By visualizing PSR-stained tissues under a red fluorescent filter, we can reveal collagen fibers with a clarity comparable to that of second harmonic generation imaging (Wegner et al., 2017b). Stained tissue sections were cleared with xylene and mounted with Richard-Allan toluene-based mounting medium (Richard Allen Scientific, San Diego, CA, USA). Tissues were imaged using an Eclipse E600 compound microscope as described in the ‘Histology and immunostaining’ section. Tissues were also imaged using a BZ-X710 digital microscope (Keyence, Itasca, IL, USA), fitted with 10× or 20× dry objectives (PlanFluor, 0.45 NA) and FITC and Texas Red filter cubes. Tiled images were captured and stitched using image acquisition software (Keyence, Itasca, IL, USA).

PSR fluorescence in mouse prostate tissues is confounded by autofluorescence of secretory material within prostate ducts and within some prostate cells. Autofluorescence was removed by subtracting pixels captured through the FITC filter from pixels captured through the Texas Red filter using the ‘image calculator’ function of ImageJ. Additional fluorescence from ductal lumens was removed manually. Total tissue area was measured by creating a freehand selection boundary around the prostate perimeter and measuring the pixel area of the selected area. Collagen density was measured by quantifying the area of PSR staining relative to total tissue area. Collagen fiber density was determined using CT-FIRE software, a plugin for ImageJ, which automatically detects and quantifies collagen features (Bredfeldt et al., 2014).

PSR-stained collagen fibers in human tissues were quantified as follows: a region of interest (ROI) selection box (5.0 mm×1.0 mm) was placed perpendicular to the urethra, centered on the longest edge. One side (1.0 mm) of the ROI box was aligned immediately adjacent to the urethra, and the box projected out from the urethra (5.0 mm) into surrounding prostate tissue. Each ROI was divided into 2200 1.0 pixel-wide (2.27 µm) columns. The average pixel brightness of each column was measured as a gray value using the ‘plot profile’ function within ImageJ (FIJI). Each column's average brightness was plotted against the column's distance to the urethra. A summary of the methodology is in Fig. S1.

### *In situ* detection of RNAs using RNAscope™ multiplex fluorescent reagent kit v2

RNAscope*™* probes against human *GLI1*, *LYZ* and *COL1A1* were acquired from Advanced Cell Diagnostics (Hayward, CA, USA). Sections were deparaffinized in xylene, rehydrated, air dried, treated with endogenous hydrogen peroxidase block solution at room temperature for 10 min, immersed in pretreatment 2 solution at 100-104°C for 15 min, and digested with protease solution for 30 min at 40°C. Slides were rinsed with distilled water twice after each step. Probes were then hybridized at 40°C for 2 h in a humidified chamber. After washing, signal amplification from the hybridized probes was performed by the serial application of amplification solutions as per the RNAscope*™* instructions. Opal dyes [Opal 520, FP1487001KT; Opal 570, FP1488001KT; Opal 690 reagent pack, FP1497001KT (Akoya Biosciences, Marlborough, MA, USA)] were reconstituted in dimethyl sulfoxide (DMSO) and diluted in tyramide signal amplification buffer (1:1000). Horseradish peroxidase (HRP)-C1 and HRP-C2 signals were developed as per the RNAscope*™* instructions, and the slides were counterstained with DAPI and coverslipped using antifade mounting medium. Slides were imaged either as described in the ‘Histology and immunostaining’ section or using a Keyence BZ-X700 microscope, with images stitched and compressed into whole images.

### Hybridization chain reaction for detection of RNA in prostate tissue sections

An HCR kit was purchased from Molecular Instruments^®^ (Eagle Rock, CA, USA). Dorsal prostate sections (5 μm) were baked in a dry oven for 1 h, deparaffinized in xylene and rehydrated with a series of graded ethanol washes (100%, 95%, 70%, 50% and water) at 25°C. Sections were immersed in 1× Tris-EDTA buffer (pH 9.0) for 15 min at 95°C, and 100 ml water was added every 5 min for 20 min to decrease the temperature to 45°C. Sections were immersed in water for 10 min and then digested with 10 µg/ml proteinase K solution (VWR, Radnor, PA, USA) in a 37°C humidified chamber for 10 min. Sections were washed in 1× PBS twice after each pretreatment step.

Sections were pre-hybridized for 10 min inside a humidified chamber with probe hybridization buffer. Probe solution was prepared by adding 0.4 pmol of each probe set to 100 µl probe hybridization buffer and added to sections and incubated overnight at 37°C in a humidified chamber. Excess probes were removed with a series of saline-sodium citrate (SSC) washes (75% probe wash buffer/25% 5× SSC, 50% probe wash buffer/50% 5× SSC, 25% probe wash buffer/75% 5× SSC, 100% 5× SSC) in a 37°C humidified chamber for 15 min each, followed by a 100% 5× SSC wash at 25°C.

Amplification buffer was added on top of the tissue, and sections were pre-amplified at 25°C for 30 min. Hairpins were prepared separately by snap cooling 6 pmol h1 hairpin and 6 pmol h2 hairpin (heat at 95°C for 90 s and cooling to room temperature in a dark drawer for 30 min). Both hairpins were added to 100 µl amplification buffer and added to sections overnight in a dark humidified chamber at 25°C. Excess hairpins were removed with 5× SSC at 25°C for 1×5 min, 2×15 min, and 1×5 min. DAPI was added to each section for 5 min, then washed at 25°C with 5× SSC 3×5 min. Sections were cover slipped with antifade mounting medium and imaged as described in the ‘Histology and immunostaining’ section.

### Reverse transcription and real-time quantitative PCR

RNA was purified with an Illustra RNAspin minikit (GE Healthcare, Pittsburgh, PA, USA) and reverse transcribed with a SuperScript III first-strand synthesis system (Invitrogen, Carlsbad, CA, USA). Real-time PCR was performed in 10.5 μl reactions containing 1× SsoFast EvaGreen Supermix (Bio-Rad, Hercules, CA, USA), 0.48 μm PCR primers and 3.75 μl cDNA, and amplified using a CFX96 PCR machine (Bio-Rad) as previously described (Keil et al., 2012). Relative mRNA expression was determined by the ΔC_t_ method and normalized to *Ppia* expression. Probes are listed in Table S4.

### Statistical analysis

Statistical analyses were performed with Graph Pad Prism 8.0.2. Differences were considered significant at the *P*<0.05 level. A Shapiro–Wilk test was used to test for normality, and transformation was applied to normalize data. Bartlett's test was used to test for homogeneity of variance. For pairwise comparisons, unpaired two-tailed Student's *t*-test was applied when variances were equal, and a *t*-test with Welsh's correction was applied when variances were unequal, between groups. A Mann–Whitney test was performed when data could not be normalized through transformation. Linear regression analysis was performed to determine the coefficient of determination (*r*^2^) and whether slopes of linear regression lines differed (Fig. 1C). For tests with two independent variables, two-way ANOVA that passed (*P*>0.05) Spearman's test for heteroscedasticity and the Shapiro–Wilk test for normality of residuals was used, followed by Sidak's multiple comparisons test (Fig. 7B).

## Supplementary Material

10.1242/dmm.052012_sup1 Supplementary information
