# Supplementary material for: Prostatic Escherichia coli infection drives CCR2-dependent recruitment of fibrocytes and collagen production
Source: Dis Model Mech. 2025 Jan 24;18(1):DMM052012. doi: 10.1242/dmm.052012 (PMC11789281; doi:10.1242/dmm.052012)
Supplement: Supplementary information [file dmm-18-052012-s1.pdf]

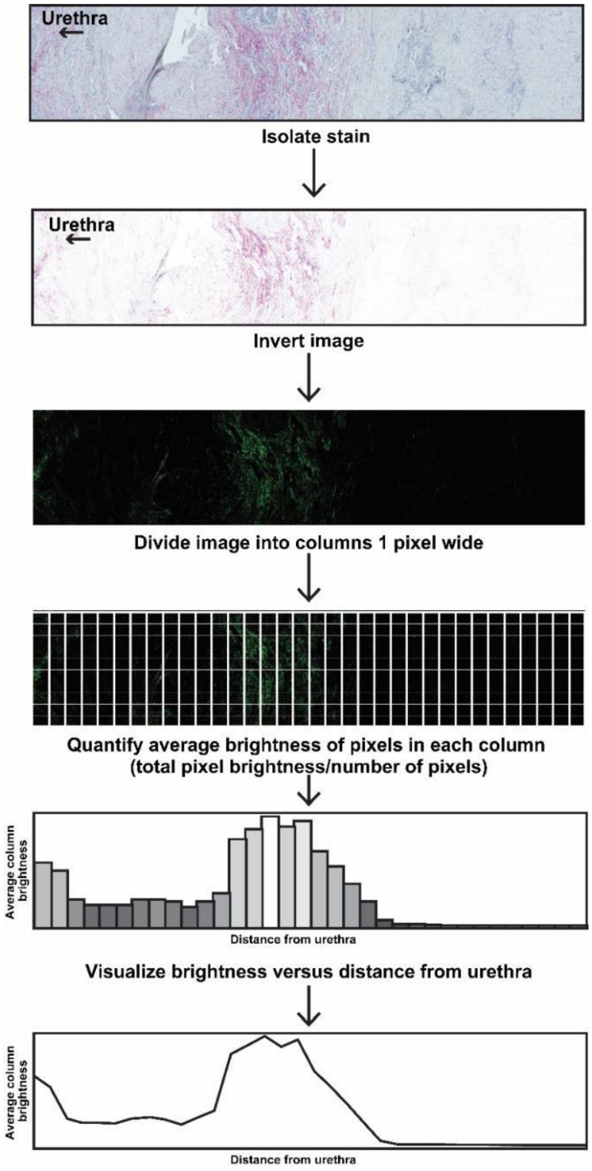

Fig. S1. Method for quantifying picrosirius red stain brightness. Prostate sections are stained and imaged using fluorescent microscopy. A 5 mm x 1 mm region of interest (ROI) is selected from the image. All subsequent steps are performed using the plot profile function of ImageJ. Images of picrosirius red fluorescence are then divided into one-pixel-wide columns. The average brightness of each column is calculated as a gray value. The sum of the gray values of all the pixels in the column are divided by the number of pixels in the column ("Analyze Menu," n.d.). The brightness is then plotted relative to location of the column to the urethra.

Disease Models & Mechanisms • Supplementary information

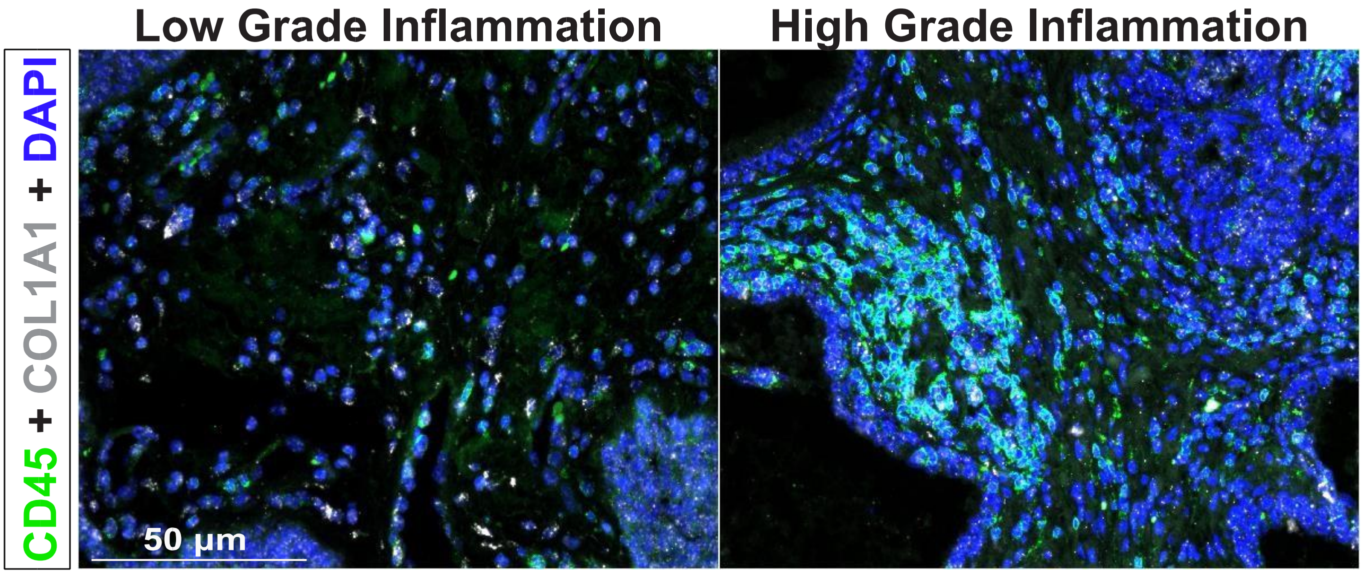

Fig. S2. Inflammation, characterized by abundance CD45+ cell (leukocyte) infiltration and *COL1A1*+ cells. IF was performed to visualize CD45 protein and the fluorescent RNAscope™ assay was used to visualize *COL1A1* RNA in the prostate transition zone. Sections were separated into two categories (A) low-grade inflammation (CD45+ and COL1A1+ cells are present but defuse) or (B) high-grade inflammation (CD45+ and *COL1A1*+ cells are present and concentrated). Images were taken using an Eclipse E600 compound microscope at 20x magnification. Sections classified as having high inflammation were used.

Disease Models & Mechanisms • Supplementary information

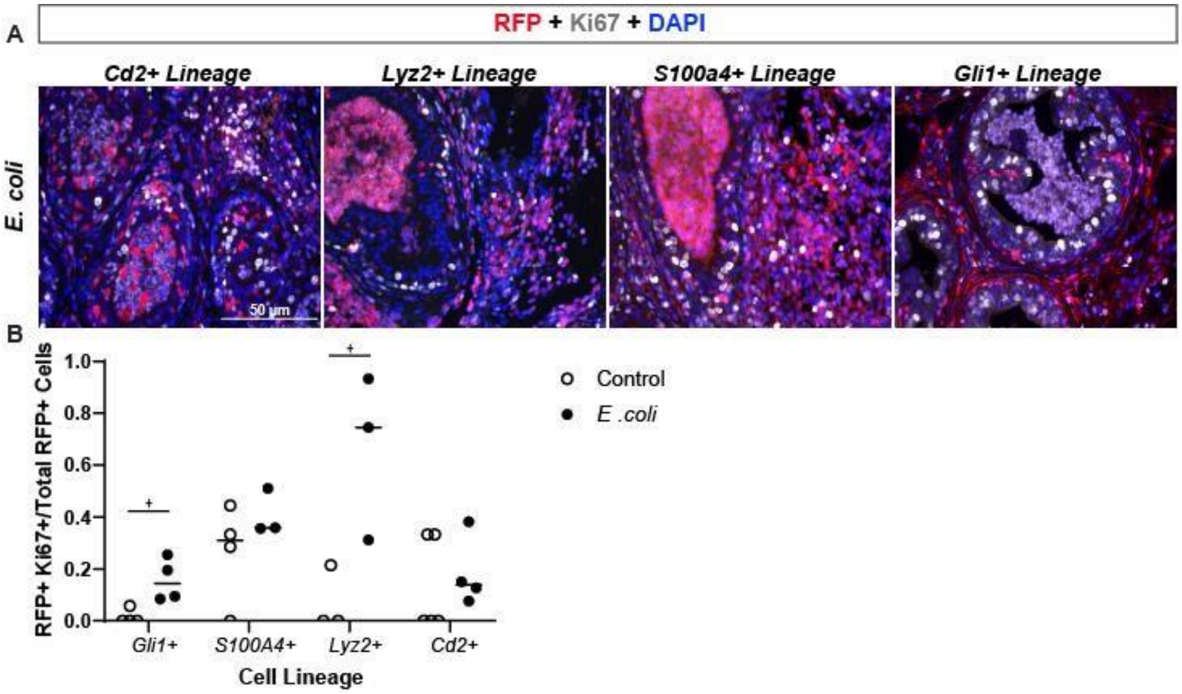

Fig. S3. Inflammation drives the proliferation of *Lyz2*+ and *Gli1*+ cell lineages but not *S100a4*+ and *Cd2*+ cell lineages. The Ai14 reporter allele was bred *Gli1-CreER*^{T2}, *Lyz2CreER*^{T2}, *S100a4-cre, and Cd2-icre* to make reporter mice. Seven-week-old *Gli1* reporter mice were administered 200mg/kg/day of tamoxifen for four days to activate *cre*; the other *cre* alleles are constitutively active. At eight weeks of age, reporter mice were transurethrally catheterized and administered two bolus doses, spaced 24 h apart, of *E. coli* UTI89 (OD 0.80 in 100 µL PBS). Mice were euthanized seven days after the first bolus dose of *E. coli*. Lower urinary tracts were collected, fixed, embedded in paraffin, sectioned and immunostained with an antibody against RFP to visualize the Ai14 reporter, an antibody against Ki67 to reveal cells in the active phases of the cell cycle, and DAPI was used to mark cell nuclei. The percentage of RFP+ cells co-expressing Ki67 was determined. Results are representative of 3-5 mice per group. For each genetic strain, the abundance of labeled cells in *E. coli* treated mice was compared to the abundance of labeled cells in PBS treated mice using a student's t-test. A $p < 0.05$ was considered statistically significant.

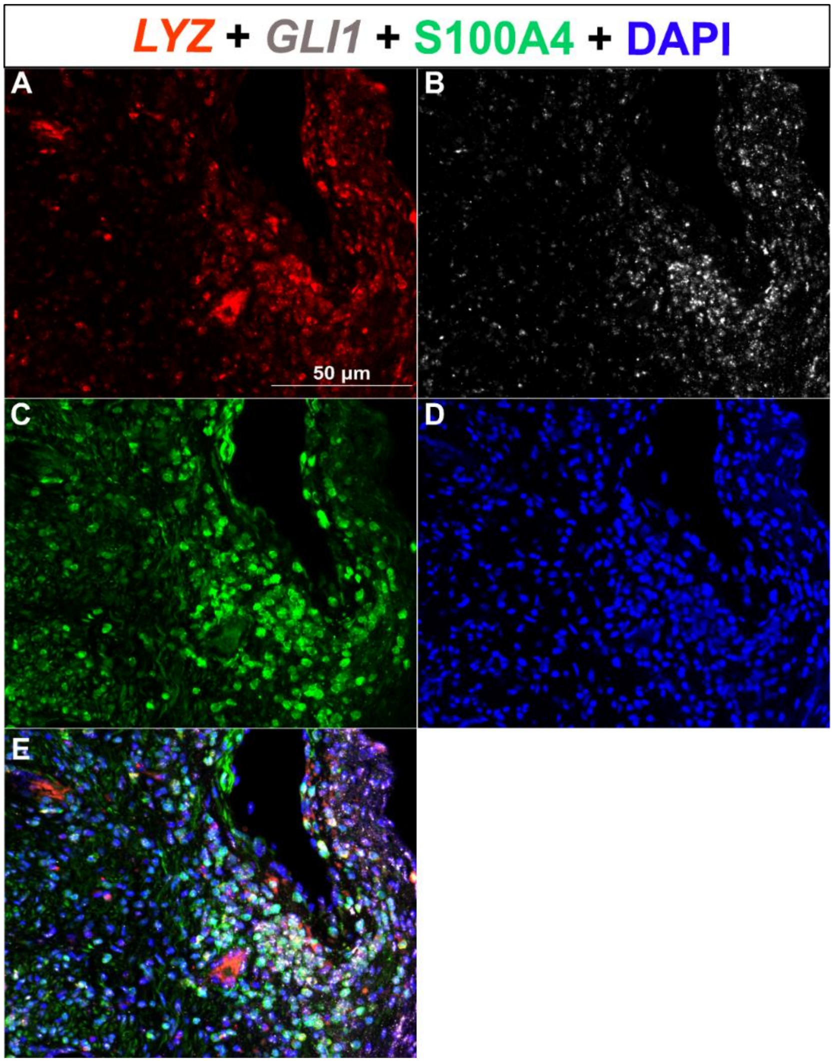

Fig. S4. Higher magnification of *LYZ*+/*GLI1*+/S100A4+ triple positive cells present in the transition zone of the inflamed human prostate, Human prostate, obtained by simple prostatectomy from men with LUTD (Supplementary Table S1.), were cut into sections and was categorized as having high-grade inflammation based on total number of CD45+ immune cells (Fig. S5). The RNAscope^{TM} Assay was used to visualize (A) *LYZ and* (B) *GLI1* mRNA, IF was used to visualize (C) S100A4 protein, and (D) DAPI to visualize nuclei. Tissues were imaged using an Eclipse E600 compound microscope at 20x. Each channel is presented separately in addition to an overlay image (E) to emphasize co-expression.

Disease Models & Mechanisms • Supplementary information

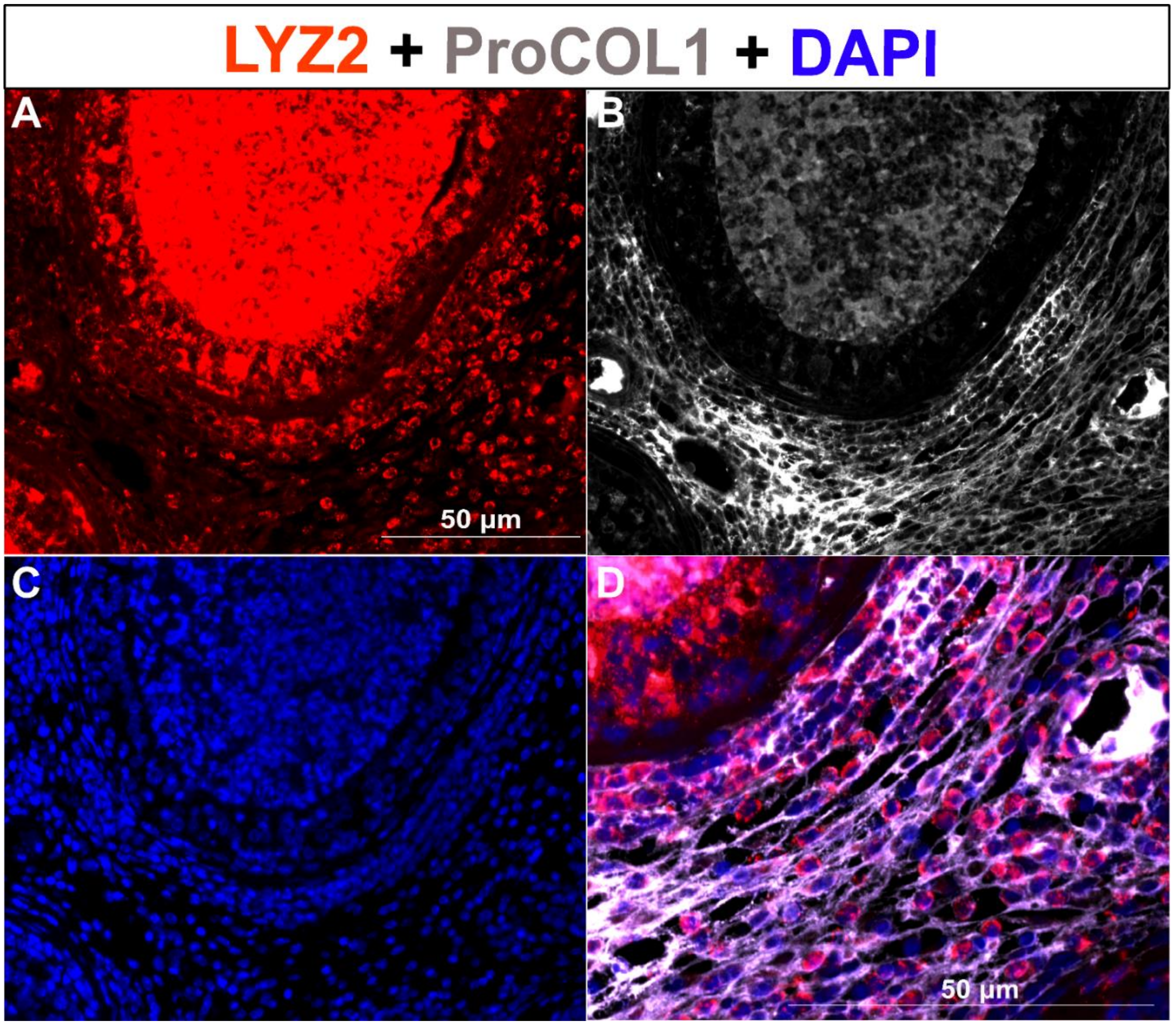

Fig. S5. Higher magnification of LYZ2+ and ProCOL1+ co-expressing cells in the *E. coli* infected CCR2$^{+/-}$ mouse prostate. Eight-week-old control (*Ccr2$^{+/-}$*) male mice were transurethrally catheterized and administered two bolus doses, spaced 24 h apart, of *E. coli* UTI89 (OD 0.80 in 100 µL PBS) or sterile PBS. Mice were euthanized seven days after the first bolus dose of *E. coli*. Ccr2$^{+/-}$ mouse dorsal prostate sections were stained with antibodies against (A) LYZ2, the intracellular collagen precursor, (B) proCOL1A1, and (C) DAPI to visualize nuclei. Each channel is presented separately to emphasize co-expression and (D) LYZ2+proCOL1+ double positive cells are shown at higher magnification. Images were taken in the dorsal prostate lobe using an Eclipse E600 compound microscope at 20x magnification.

Disease Models & Mechanisms • Supplementary information

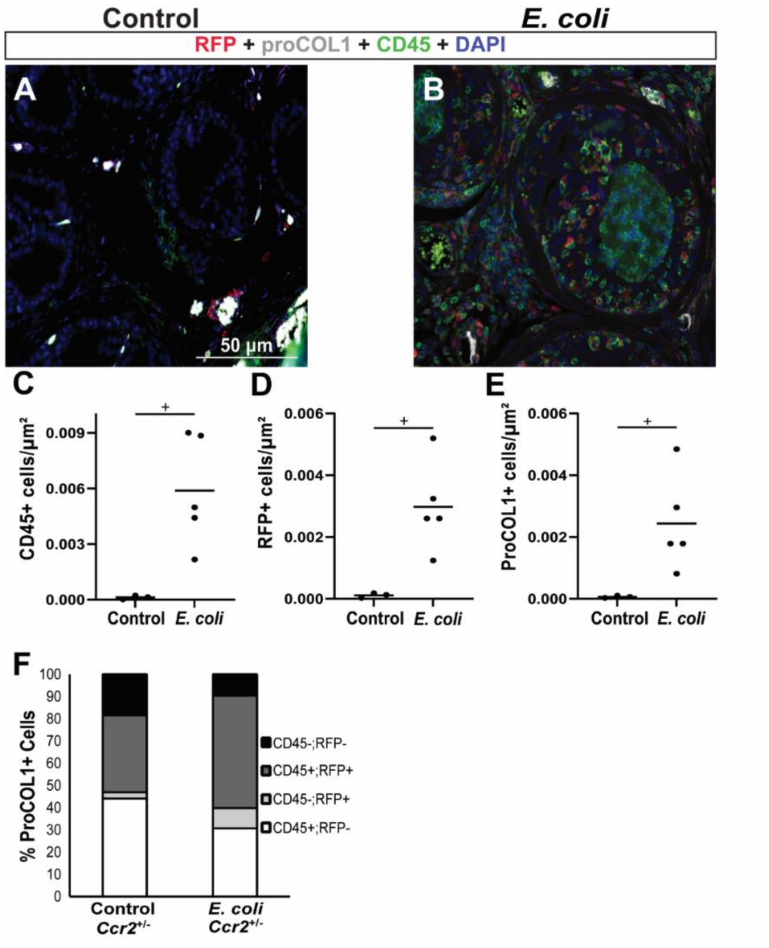

Fig. S6. *Ccr2*+ cells are recruited to the inflamed mouse prostate. Eight-week-old *Ccr2*$^{+/-}$ mice were transurethrally catheterized and administered two bolus doses, spaced 24 h apart, of *E. coli* UTI89 (OD 0.80 in 100 µL PBS) or sterile PBS (control). Mice were euthanized seven days after the first bolus dose of *E. coli*. Lower urinary tracts were collected, fixed, embedded in paraffin, sectioned and immunostained with antibodies against CD45 (leukocyte marker), Procollagen 1 (ProCOL), and RFP (A & B). DAPI was used to mark cell nuclei. Percentages of ProCOL+ cells coexpressing CD45, RFP, or both were determined (C-F). Results are representative of 3-5 mice per group. Groups were compared using a student's t-test. A $p < 0.05$ was considered statistically significant.

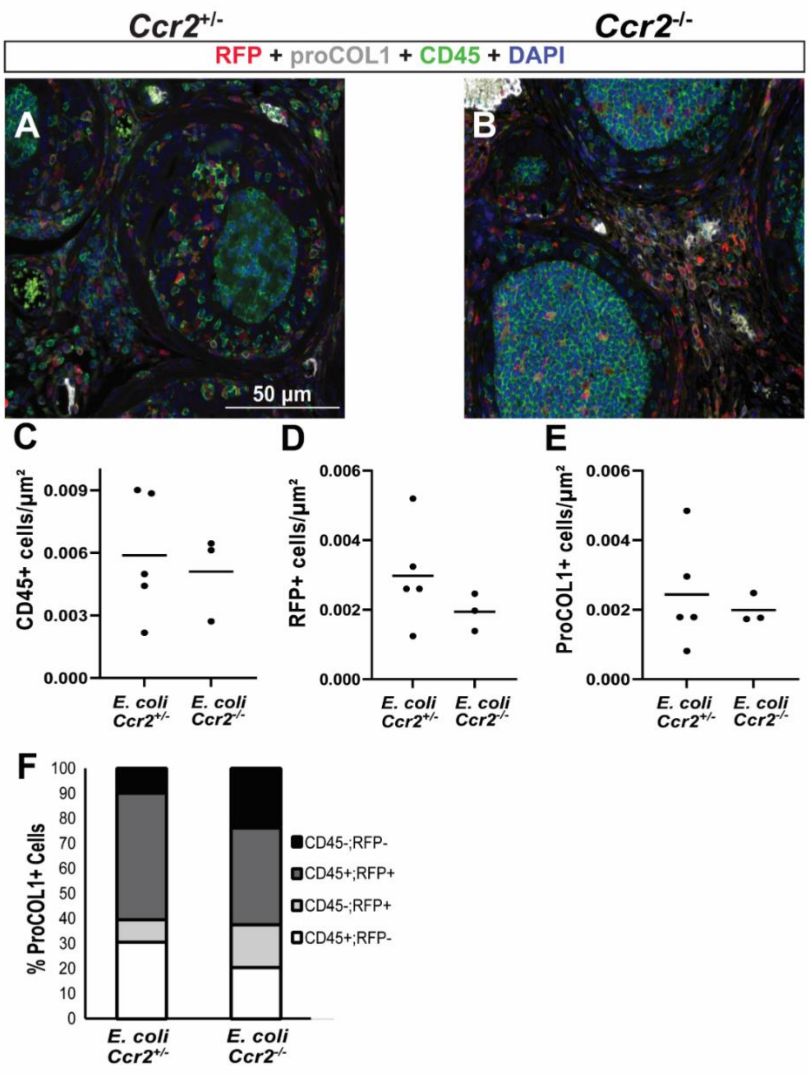

Fig. S7. *E. coli* drives prostate inflammation in *Ccr2* null mice. Eight-week-old *Ccr2*$^{-/-}$ mice were transurethrally catheterized and administered two bolus doses, spaced 24 h apart, of *E. coli* UTI89 (OD 0.80 in 100 µL PBS. Mice were euthanized seven days after the first bolus dose of *E. coli*. Lower urinary tracts were collected, fixed, embedded in paraffin, sectioned and immunostained with antibodies against CD45 (leukocyte marker), Procollagen 1 (ProCOL), and RFP (A & B). DAPI was used to mark cell nuclei. Percentages of ProCOL+ cells co-expressing CD45, RFP, or both were determined (C-F). Results are representative of 3-5 mice per group. Groups were compared using a student's t-test, panels C-E were not significant. A $p < 0.05$ was considered statistically significant.

Disease Models & Mechanisms • Supplementary information

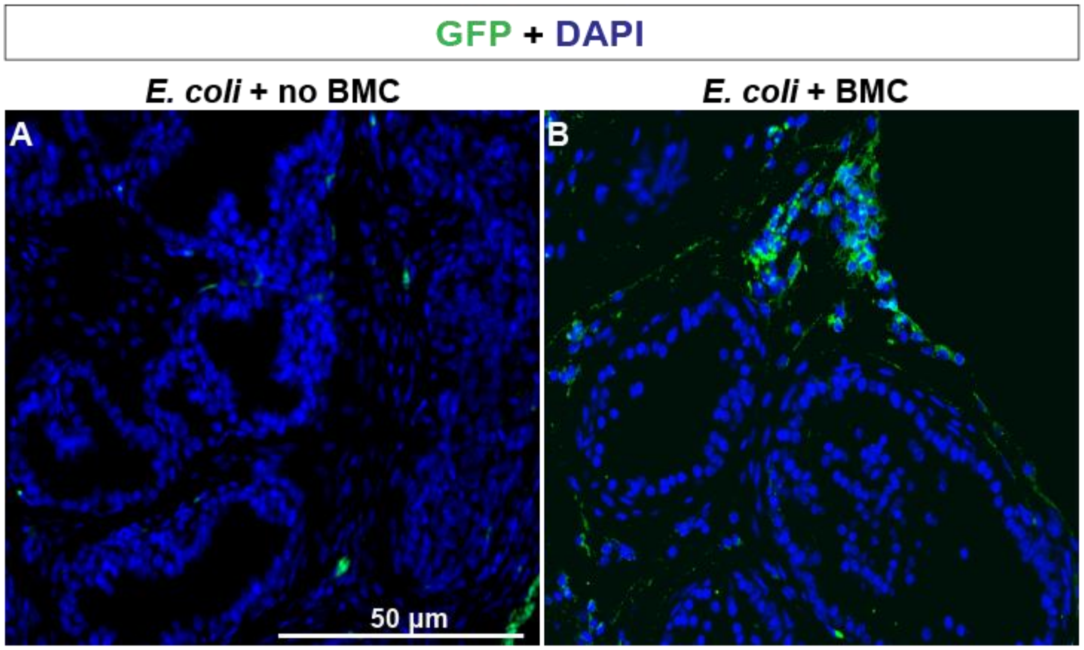

Fig. S8. GFP retroorbital injected donor cells migrate to the *E. coli* instilled *Ccr2*$^{-/-}$ mouse prostate. Eight-week-old *Ccr2*$^{-/-}$ mice were anesthetized with isoflurane and were retro-orbitally injected with (A) PBS alone or (B) bone marrow cells from Rosa-GFP donor mice (27 ga. needle, 5 million cells in 100 µl phosphate buffered saline, PBS). Mice were immediately transurethrally catheterized and administered two bolus doses, spaced 24 h apart, of *E. coli* UTI89 (OD 0.80 in 100 µL PBS). IF was performed to visualize GFP protein and DAPI was used to visualize nuclei. There was some non-specific background staining immunopositive for GFP in the PBS alone (A), however no nucleated cells were positive validating that our staining in (B) is accurately staining our injected bone marrow cells from Rosa-GFP donor mice. Images were taken using an Eclipse E600 compound microscope at 20x magnification.

Disease Models & Mechanisms • Supplementary information

Table S1. Human prostate tissue sample information.

Donor ID	Age	Ethnicity	Figure
D05	Unknown	Unknown	1
D06	47	Hispanic/Latino	1
D07	19	White	1
D12	Unknown	Unknown	1
D18	25	Asian	1
D20	18	White	1
D22	38	Hispanic/Latino	1
D35	29	White	1
D45	Unknown	Unknown	1
D54	34	White	1
**BPH ID**	**Age**	**Ethnicity**	**Figure**
BPH 02	65	Hispanic/Latino	1
BPH 211	70	Asian	1
BPH 224	75	White	1
BPH 230	82	White	1
BPH 262	67	White	1
BPH 307	68	White	5
BPH 327	74	White	1
BPH 331	59	Asian	5
BPH 334	70	Hispanic/Latino	5
BPH 339	68	Asian	1
BPH 357	Unknown	Hispanic/Latino	1
BPH 380	67	White	1
BPH 381	68	White	1
BPH 389	67	Asian	1
BPH 405	74	White	S2(A)
BPH 409	66	White	1
BPH 412	81	White	1
BPH 416	67	Asian	1
BPH 420	66	White	1
BPH 421	74	Hispanic/Latino	1
BPH 424	76	Hispanic/Latino	2, 5
BPH 428	82	Black/African American	1
BPH 430	83	White	2
BPH 440	64	Hispanic/Latino	S2
BPH 444	67	Hispanic/Latino	S2
BPH 455	66	White	S2
BPH 461	75	White	2
BPH 494	67	White	5, S2(B)

Table S2. The combined contributions of *Gli1*+, *Lyz2*+, *CD2*+ and *S100a4*+ lineages to prostatic collagen producing cells exceeds 100%.

Cell Lineage	Contribution to Total Collagen Producing Cells in *E. coli* Infected Prostate (% RFP+ProCOL1A1 / Total ProCOL1A1)
Gli1+	70.0%
S100A4+	86.3%
Lyz2+	70.6%
CD2+	27.5%
**SUM**	**>100%**

Table S3. Antibodies used for immunostaining.

Name(s)	Symbol	Species	Antibody Registry (RRID)	Supplier	Catalog Number	Dilution
Smooth Muscle Actin Alpha	Acta2	Mouse	AB_442134	Leica	Ncl-Sma	1:250
protein tyrosine phosphatase, receptor type C	CD45	Rabbit	AB_ 442810	Abcam	Ab10558	1:500
Red Fluorescent Protein	RFP	Goat	AB_2722750	Sicgen	AB8181-200	1:500
Procollagen 1	ProCOL	Mouse	AB_528438	Developmental Studies Hybridoma Bank	SP1.D8	1:1000
S100 calcium binding protein A4	S100A4	Rabbit	AB_2183775	Abcam	Ab27957	1:100
Green Fluorescent 0Protein	GFP	Chicken	AB_300798	Abcam	Ab13970	1:200
Antigen identified by monoclonal antibody Ki 67	KI67	Mouse	AB_15580	Abcam	Ab15580	1:300
Anti-Rabbit 488		Rabbit	AB_2313584	Jackson ImmunoResearch	711-545-152	1:250
Anti-Mouse 647		Mouse	AB_2313584	Jackson ImmunoResearch	715-605-150	1:250
Anti-Rabbit 647		Rabbit	AB_2492288	Jackson ImmunoResearch	711-605-152	1:250
Anti-Chicken 488		Chicken	AB_2340376	Jackson ImmunoResearch	703-546-155	1:250
Anti-Goat RRX		Goat	AB_2340423	Jackson ImmunoResearch	705-295-147	1:250

Table S4. RT-PCR primers for measuring *Ccl2* and *Ppia* mRNA abundance in mouse dorsal prostate.

Target NM Number	Left Primer	Right Primer
Ccl2 NM_011333.3	TGACCCGTAAATCTGAAGCTAATG	TCACTGTCACACTGGTCACTCC
Ppia NM_008907.2	TCTCTCCGTAGATGGACCTG	ATCACGGCCGATGACGAGCC
